# Enzyme Immobilization

**DOI:** 10.3390/molecules28031373

**Published:** 2023-02-01

**Authors:** Roberto Fernandez-Lafuente

**Affiliations:** Departamento de Biocatálisis, ICP-CSIC, Campus UAM-CSIC, 28049 Madrid, Spain; rfl@icp.csic.es; Tel.: +34-91594804

The development of enzyme immobilization started in the middle of the previous century as a potential answer to the problem of the enzyme recovery and reuse [1]. These biocatalysts were very expensive at that time and their single use could only be performed on very high added value products or in academia. Nowadays, the price of enzymes has decreased and some enzymes are commercialized for one use even in moderately cheap product production (e.g., Eversa to produce biodiesel [2]). However, immobilization has many positive effects that can justify its development and use. Together with enzyme reuse, immobilization can improve enzyme stability for different reasons: broadening the enzyme operation window [3,4], improving enzyme activity, selectivity or specificity [5,6] and even becoming coupled to enzyme purification [7]. That way, immobilization remains as an important tool in the design of industrial biocatalysts [8]. Moreover, far from being a mature discipline, many of the factors that determine the immobilized enzyme performance still remain unsolved [8]. A proof of the interest and potential of enzyme immobilization is the fact that many Special Issues in MDPI journals in 2022 or those still open in 2023 are related in some sense with this objective. Among them is this Special Issue, Enzyme Immobilization IV. It is the fourth issue on this topic that I have edited in *Molecules*. In this new issue, 10 papers have been collected.

Many of the contributions published in this issue are related to the immobilization of lipases, perhaps the most used enzyme family in biocatalysis [9,10]. In the first one, Guimarães et al. show the way in which the immobilization of Eversa in the form of magnetic cross-linked enzyme aggregate transform the enzyme in a suitable biocatalyst for the transesterification of waste cooking oil with different alcohols, producing valuable biolubricants, when the free enzyme was very poorly efficient for this goal [11]. A second paper shows the possibility of modulating the properties of a lipase from the extremophilic microorganism Serratia sp. USBA-GBX-513 by using different immobilization protocols [12]. This lipase modulation has been the object of many different publications [5,6], but there are not many papers describing immobilization of enzymes from extremophiles [13]. Another paper exemplifies that enzyme immobilization may be compatible with any other enzyme modulation strategy [14]. In this case, the immobilized commercial lipases Lipozyme^®^TL (TLL-IM) (lipase from *Thermomyces lanuginosus*), Lipozyme^®^435 (L435) (lipase B from *Candida antarctica*), Lipozyme^®^RM (RML-IM), and LipuraSelect (LS-IM) (both from lipase from *Rhizomucor miehei*) were submitted to mineralization processes [15], in a similar form to the preparation of nanoflowers using free enzymes [16]. This modification permitted to employ the benefits of enzyme mineralization (changes in activity and enantiospecificity in these examples) [15] without the problems derived of the small size and fragile nature of nanoflowers [16]. Another paper uses the commercial immobilized lipase Lipozyme 435 to produce xylose oleate in methyl ethyl ketone from xylose and oleic acid [17]. The last paper, using only lipases, shows the enzymatic synthesis of ascorbyl palmitate catalyzed by the commercial immobilized lipases Amano Lipase PS, Lipozyme^®^ TL IM, Lipozyme^®^ Novo 40086, Lipozyme^®^ RM IM and Lipozyme^®^ 435, selecting Lipozyme^®^ 435 for further studies [18]. Using 2-methyl-2-butanol as solvent, the global results could be improved, and the biocatalyst was used in a basket reactor with very good results (yields remained over 80% after four sequential batches).

Five immobilized lipases are also some of the examples of the paper from Braham et al., where it was shown that the inactivation conditions and immobilization protocol determine the intensity and sense effects of some salts on enzyme stability [19]. This paper uses other enzymes as examples of this complex effect of the salts on the immobilized enzyme stabilities: three proteases, two glycosidases, and one laccase, penicillin G acylase and catalase.

The enzyme β-galactosidase is the second most utilized enzyme in this Special Issue. In the first example, the enzyme is immobilized and stabilized by immobilization on gold nanoparticles modified with polyvinyl alcohol [20]. Another paper shows the β-galactosidase immobilization on a *Bacillus subtilis* spore [21]. The authors introduce the spore divergent cohesin modules that can specifically bind to the target enzyme bearing the matching dockerins. The paper shows the results obtained utilizing five different pairs of cohesins and dockerins. The last paper on this enzyme family shows biomineralization strategy for the formation of hybrid nanocrystals from β-galactosidase [22]. An important effect of metal ions and pH on the immobilization yield and the recovered activity was determined. In silico studies identified the ion binding sites under the different conditions. The synthesis of galacto-oligosaccharides was accomplished with these biocatalysts.

The last paper is on the immobilization of penicillin G acylase [23,24] on vinyl sulfone activated supports [25]. These supports have been recognized recently as very well suited to yield intense multipoint covalent attachment [3], and the enzyme had previously immobilized/stabilized on glyoxyl and epoxy supports [26,27]. However, the immobilization failed on vinyl sulfone agarose beads. The authors were able to force the enzyme immobilization using high ionic strength and enabling the hydrophobic enzyme adsorption on the moderately hydrophobic support surface, achieving very good stabilization results after optimization of the multi-point covalent immobilization [25].
